# Exploring the Role of Advanced Practice Nurses in Cardiology: A Scoping Review

**DOI:** 10.1111/inr.70054

**Published:** 2025-06-29

**Authors:** Marco Sguanci, Stefano Mancin, Viviana Carù, Niccolò Simonelli, Giovanni Cangelosi, Sara Morales Palomares, Gaetano Ferrara, Alessio Lo Cascio

**Affiliations:** ^1^ Polyclinic San Martino Hospital Genoa Italy; ^2^ IRCCS Humanitas Research Hospital Rozzano Milan Italy; ^3^ Department of Cardiology Circolo e Fondazione Macchi Hospital ASST Sette Laghi Varese Italy; ^4^ Department of Translational Medicine University of Piemonte Orientale Novara Italy; ^5^ SS Antonio e Biagio e C.Arrigo University Hospital Alessandria Italy; ^6^ Units of Diabetology ASUR Marche Fermo Italy; ^7^ Department of Pharmacy Health and Nutritional Sciences (DFSSN) University of Calabria Rende Italy; ^8^ Nephrology and Dialysis Unit Ramazzini Hospital Carpi Italy; ^9^ Department of Biomedicine and Prevention Tor Vergata University Rome Italy

**Keywords:** advanced practice nurse, cardiology, clinical nurse specialists, nurse practitioner, review

## Abstract

**Aim:**

To analyze the role of advanced practice nursing (APN) in the cardiology context in outpatient and inpatient settings, detailing their roles, competencies, and responsibilities. It also provides an overview of the main degrees and postgraduate training programs for specialization in cardiology management in major Western countries.

**Background:**

The increasing complexity of the healthcare sector requires an evolution in nursing education and clinical competencies to effectively manage care in complex and interdisciplinary contexts such as cardiology settings.

**Methods:**

This scoping review followed the Joanna Briggs Institute framework. The Preferred Reporting Items for Systematic Reviews and Meta‐Analyses extension for Scoping Reviews (PRISMA‐ScR) was used to ensure rigorous and transparent reporting.

**Results:**

Among the 647 records analyzed, 15 were included in this review. In the cardiology setting, the APN plays a critical role in clinical care, education, and research, providing both direct and indirect support to patients at all stages, across outpatient and inpatient settings. Thanks to the advanced skills acquired through specific paths, it also acts as a link between the healthcare team and patients, improving the provision of care through personalized and coordinated actions and promoting continuous training and research in the field.

**Conclusions:**

APN enhances cardiac care by offering in‐depth clinical assessments, education, and support throughout the treatment process. However, the lack of standardized regulatory frameworks in some countries limits the autonomy of cardiology APNs within the healthcare system, compromising their ability to address unmet healthcare needs and collaborate internationally.

## 1 Introduction

Cardiovascular diseases (CVD) are the world's topmost killer (WHF 2013); nearly 18 million people die each year from these diseases, which accounts for approximately 31% of all global deaths: of these, 85% result from heart attacks and strokes (WHO 2017).

The conditions of the heart or blood vessels, such as heart attack, stroke, and heart failure, kill 20.5 million every year, mostly in low‐ and middle‐income countries (Piñeiro et al. 2023).

However, many of these conditions are preventable and manageable by addressing behavioral risk factors (Di Cesare M. et al. 2024): individuals diagnosed with CVD or those at high risk need early diagnosis and appropriate management through counseling and pharmacotherapy (Obeidat et al. 2023). The “Global Action Plan for the Prevention and Control of Noncommunicable Diseases 2020–2030” aims to reduce premature mortality from noncommunicable diseases (NCDs) such as CVD by 25% by 2025. This plan emphasizes preventive strategies, risk factor reduction, and scaling up control measures through people‐centered healthcare, promoting high‐quality research, and monitoring trends in NCD prevention and control. It also includes initiatives to strengthen human resources by improving the knowledge, skills, and motivation of health workers, as well as supporting career development through advanced training in areas such as nursing and optimizing nurses’ roles to contribute effectively to the prevention and control of NCDs (WHO 2013).

Advanced practice nursing (APN) represents an advancement in assistance that benefits patients, healthcare systems, and the nursing profession (Hansen et al. 2022). This role is rooted in a foundation of advanced clinical knowledge, and experience, based on advanced specialist education that aims to acquire additional competencies through specialized training and operating within a broader scope of nursing practice as the characteristics are shaped by the specific context of accredited competence (Burton et al. 2012; International Council of Nursing 2020). These skills are essential to enable nurses to manage care in complex environments, embracing an interdisciplinary perspective that spans all health and social sectors (Bayot and Varacallo 2024).

The two main roles identified for APNs are clinical nurse specialist (CNS) and nurse practitioner (NP) (International Council of Nursing 2020). The concept of APN has evolved over time, with the first nurse specialists emerging in the 19th century, followed by the development of CNSs in the 1960s: In the United States, four distinct categories of APNs have been identified, one of which is the CNS (Hamric et al. 2014). In the United Kingdom, CNSs are classified as a subset of advanced nurse practitioners (ANPs) (Cooper et al. 2019), while in Ireland CNSs are considered a lower level than ANPs (Begley et al. 2010).

In recent years, the global demand for APNs has significantly increased, particularly in several Western countries, where their numbers have been growing at a faster rate than those of physicians (Maier et al. 2016). APNs are capable of delivering high‐quality care that is comparable with that provided by physicians across a wide range of acute and chronic conditions. They represent an effective and efficient resource to address the challenges of delivering accessible, safe, and affordable healthcare (International Council of Nursing 2020; Mancin et al. 2024).

As a result, the role of the APN is expanding in various areas of clinical care, including cardiology. Cardiovascular (CV) nursing specialization encompasses a wide range of interventions aimed at both the prevention and the treatment of cardiovascular conditions. In Europe, CV CNSs are responsible for clinical documentation, administering prescribed treatments, conducting diagnostic tests, and managing patients’ symptoms (Grešš Halász et al. 2021).

Practice nurses also play a crucial role in CV risk factor modification programs, such as smoking cessation and weight management, in addition to tasks like medication titration, screening, and conducting health assessments (Halcomb et al. 2007). Many countries around the world are increasingly integrating and recognizing the role of APNs within CV nursing practice (Smith 2015).

However, there are numerous diverse professional and educational pathways globally, which makes it challenging to establish a standardized role with consistent skills and responsibilities. This variation in training and career development leads to a lack of clarity about advanced nursing practice, with practice boundaries and career pathways that may be ambiguous, thereby causing confusion among policymakers, healthcare professionals, and the general public (International Council of Nursing 2020; Wheeler et al. 2022).

### 1.1 Aim

This scoping review aims to analyze the role of the APN in cardiology, including their functions and responsibilities, and provide an overview of qualifications and postgraduate programs for cardiology specialization.

## 2 Methods

### 2.1 Protocol and Registration

This scoping review adhered to a protocol that was prospectively registered on the Open Science Framework https://doi.org/10.17605/OSF.IO/CEWF8) and followed the Joanna Briggs Institute (JBI) approach (Peters et al. 2020a; Pollock et al. 2021). To enhance the rigor of the study, adherence to the Preferred Reporting Items for Systematic Reviews and Meta‐Analyses extension for Scoping Reviews (PRISMA‐ScR) (Tricco et al. 2018) was maintained.

### 2.2 Formulation of the Research Question

The research question for this scoping review was developed using the PCC framework (Peters et al. 2020). The PCC framework assists authors in developing a research question for the review and is based on the following key concept: population (P), concept (C), and context (C). Based on this methodology, three aspects were included in this work: P: APN; C: exploration of the role and main postgraduate titles and training programs; C: cardiology setting.

### 2.3 Eligibility Criteria

The inclusion criteria for this scoping review were: studies investigated the role of the APN in cardiology; available in full text; English language; documents from scientific societies or scientific literature that illustrate the principal degrees and postgraduate training programs dedicated to specialization in cardiology management. The exclusion criteria were studies pertaining to book or book chapters, congress abstract articles, and articles not available in full text.

### 2.4 Search Strategy

The search was conducted in July 2024, in four databases: PubMed/Medline, Embase, CINAHL, and the Cochrane Library. The formulation of search strings for this study involved the use of keywords derived from the database thesaurus, including controlled vocabulary along with additional keywords and Boolean operators. Keywords identified were “Advanced Practice Nurse” and “Cardiology” and their variants combined appropriately with the Boolean operators AND–OR, specifically selected based on the established eligibility criteria for the study. To ensure a comprehensive exploration of available literature, gray literature was consulted to retrieve potential additional documents from the gray literature, adding depth and inclusivity to the scoping review. All records considered potentially relevant were imported into EndNote 20. Duplicate records were removed through an automated search followed by a manual search (Sguanci et al. 2024). The screening of the identified records was conducted by two academic researchers (NS and VC) with expertise in cardiology. Any discrepancies were initially addressed and, in case of disagreement, resolved through collaboration with a third academic researcher (SM). Moreover, in line with the JBI framework (Peters et al. 2020a; Tricco et al. 2018), we planned to examine the references and citations of full‐text documents obtained (Table S1).

### 2.5 Data Extraction Process

To facilitate the data extraction process to address the research questions and achieve the objectives of this scoping review, a data extraction table was developed following the JBI scoping review methodology (Peters et al. 2020a). The data extraction was performed by two academic researchers (NS and VC) with expertise in cardiology, and any discrepancies were initially addressed and, in case of disagreement, resolved in collaboration with a third academic researcher (SM). The data extracted from the included studies covered a broad range of information, systematically organized to meet the research objectives. Details included the author information, year of publication, country, study design, sample, setting, objectives, key findings, clinical competencies, and training/educational aspects.

### 2.6 Results Synthesis

The synthesis of the findings adopted a narrative approach. The results were categorized into specific clinical settings regarding the APN's role in the cardiology context. A section was then conducted on training pathways and educational programs in a cardiology setting. The findings were presented in a narrative form, supplemented by charts and tables where relevant.

## 3 Results

**FIGURE 1:**
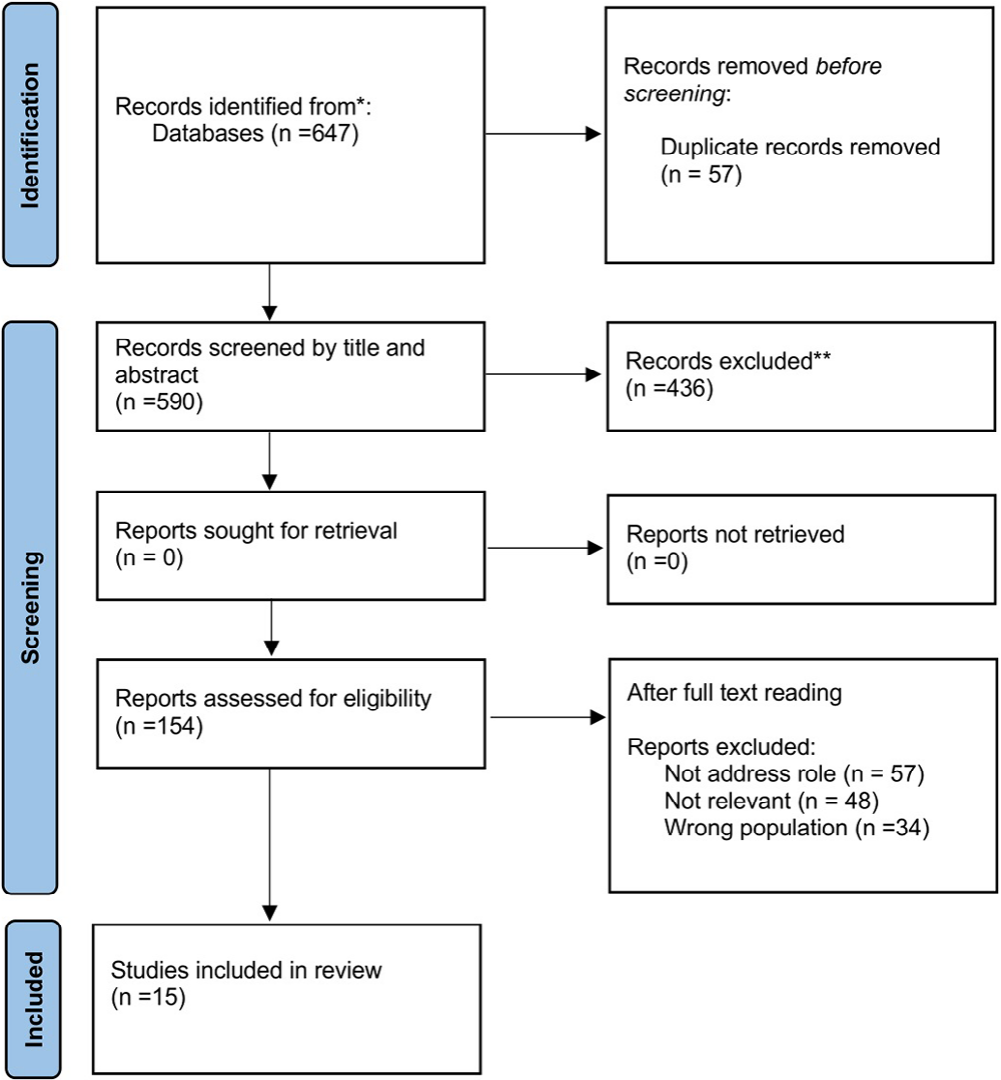
Prisma‐ScR flowchart of the process of article inclusion.

This scoping review was based on the analysis of 647 articles, selected through consultation of recognized databases such as PubMed, Cochrane Library, CINAHL, and EMBASE, as well as through examination of gray literature. From the review of titles and abstracts, only 154 studies were deemed suitable; subsequently, a detailed examination of the full texts led to the final inclusion of 16 articles. The remaining articles, examined by reading the full texts and not included in the analysis, did not address the role of the APN (*n* = 57), were not relevant to this research (*n* = 48), or were focused on the wrong population (*n* = 34) (Figure 1).

### 3.1 General Characteristics of the Studies Included

In this review, many studies have analyzed the role of various specialized professionals in the cardiology field, evaluating different aspects of their interaction and responsibilities. The articles were published between 1997 and 2022. The geographical origin of the publications came all from Anglo‐Saxon countries (the United Kingdom, Ireland, Canada, and the United States), where the figure of the CNS has now been present for several years. The study designs of the included articles were: a single randomized clinical trial (Houle et al. 2011), a qualitative study (Griffiths 2006), two cross‐sectional studies (O'Toole et al. 2019; Ingram et al. 2017), two case studies (Ingram and Khan 2014; Daniels 2014), six observational/descriptive studies (Connolly et al. 2021; Charteris and Pounds 2020; Stuart 2017; Ingram 2014; Kilpatrick et al. 2012; Boulton et al. 1997), a cohort study (Albert et al. 2008) and two narrative review (King‐Dailey et al. 2022; Johnson 2011). Specifically, of the 15 articles included in our review, five (Connolly et al. 2021; O'Toole et al. 2019; Stuart 2017; Daniels 2014; Boulton et al. 1997) were categorized as studies on the role of ANP, highlighting their effectiveness in managing patient flow and reducing hospital admissions. Three other articles (O'Toole et al. 2019; Daniels 2014; Houle et al. 2011) focused on nurse collaboration, demonstrating how collaborative care models improved the management of non‐acute chest pain. Furthermore, four articles (King‐Daley et al. 2022; Charteris and Pounds 2020; Houle et al. 2011; Nancy et al. 2010) were dedicated to heart failure management, showing how the use of telemedicine and support from specialized nurses can enhance patients’ quality of life and reduce hospitalizations. Two articles focused on therapy adherence, correlating the presence of ANP with increased adherence to recommended therapies. Finally, a study (Ingram and Khan 2014) addressed discharge protocols, highlighting the importance of an active discharge process to increase scheduled discharge rates. This research encompasses various specialties in cardiology, including clinical cardiology (King‐Daley et al. 2022; Ingram et al. 2017; Nancy et al. 2010), interventional cardiology (Boulton et al. 1997), pediatric cardiology (Daniels 2014), geriatric cardiology (Houle et al. 2011), telemedicine in cardiology (King‐Daley et al. 2022; Johnson 2011), patient education and management (O'Tolle et al. 2019; Ingram 2014), and multidisciplinary care (Ingram and Khan 2014). This review identified various roles for the APN, including CNS, NP, ANP, and physician assistant (PA) (Table S2), a medical professional who works under the supervision of a physician and is involved in diagnosing and treating patients. To provide a detailed and comprehensive overview of the roles and competencies of APNs that emerged from these studies, specific intervention areas were identified where this professional plays a central role in the field of cardiology (Table 1). The specialized competencies were analyzed and divided into the four pillars of advanced practice: advanced clinical practice; leadership; education and learning facilitation; and evidence, research, and development in accordance with the Standards for Advanced Level Nursing Practice reported by the Royal College of Nursing in 2018 (RCN 2018).

**TABLE 1:** Characteristics of included studies.

							Competencies			
Author, year	Country	Study design	Sample	Care setting	Objective	Key findings	Advance clinical practice	Leadership	Facilitation of education and learning	Evidence, research, development
Boulton et al., 1997	UK	Nonrandomized retrospective study	Patient undergoing cardiac catheterization (*n* = 200)	Cardiac center	Role exploration	CNS ensures satisfactory diagnostic images and shorter procedure times (*p* < 0.05).	√			
Griffiths, 2006	Canada	Qualitative study	Clinical nurse specialist (*n* = 4)	Hospital heart center	Role exploration	The nurse practitioner role lacks formal outcome measurement.	√	√		
Nancy et al., 2010	USA	Cohort study	Patients with chronic heart failure (HF) and LVEF <35% (*n* = 15,381)	Outpatient cardiology practices	APN influence assessment	Practices with >2 APNs/PAs show better adherence to ICD therapy and HF education (*p* < 0.01).	√		√	
Johnson, 2011	USA	Narrative review	Acute care nurse practitioner (ACNP) (*n* = 200)	University medical center, division of cardiology	Role's exploration	CNS provides quality care and professional satisfaction in cardiology.	√	√		
Houle et al., 2011	Canada	Randomized controlled trial	Patients hospitalized for ACS (*n* = 65)	University hospital	Intervention program evaluation	The experimental group showed greater physical activity at 6, 9, and 12 months.	√		√	
Kilpatrick et al., 2012	Canada	Descriptive multiple‐case study	Nurse practitioners (*n* = 59)	University hospital	Role's exploration	Competencies are adapted to the context; leadership and workload impact role effectiveness.	√	√	√	
Daniels, 2014	USA	Case study	Obstetric patient with cardiac arrhythmia (*n* = 1)	Regional clinic	Role's exploration	CNS facilitates team communication, improves patient experience, and clarifies care delivery.	√	√		
Ingram and Khan 2014	Ireland	Case study	NA	General cardiology clinic	Planning process evaluation	CNS increased discharge rates (11% to 34%, *p* < 0.0001).	√	√	√	
Ingram, 2014	Ireland	Descriptive study	NA	NA	Professional pathway exploration	CNS drives changes in healthcare services based on patient needs.	√			
Ingram et al., 2017	Ireland	Cross‐sectional study	Chest pain patients (*n* = 1041)	Outpatient chest pain clinic	Outcomes assessment	CNS identifies more elderly patients and stable coronary artery disease cases than ED physicians.	√	√	√	√
Stuart et al., 2017	UK	Descriptive phenomenological study	Nursing and medical staff (*n* = 10)	Hospital	Staff perceptions assessment	CNS provides team stability, but concerns about ANP causing de‐skilling of medical staff.	√	√	√	
O'Toole et al., 2019	Ireland	Cross‐sectional study	Patients with non‐acute coronary syndrome (*n* = 117)	Hospital	Patients’ experiences and satisfaction evaluation	ANP referral accelerates diagnosis, improves patient satisfaction, and enhances health education.	√	√	√	
Charteris and Pounds, 2020	USA	Descriptive study	HF patients (*n* = 33)	Health care center	Description of quality improvement project	HF clinic improves timeliness and patient satisfaction and reduces readmission rates (0.2%).		√	√	
Connolly et al., 2021	UK	Descriptive study	NA	Outpatient center of excellence for transcatheter mitral valve interventions	Role's exploration	CNS improves care continuity and process efficiency and reduces waiting times.	√	√		√
King‐Daile et al., 2022	USA	Narrative review	NA	NA	Clinical skill investigation	NPs collaborate to ensure optimal care for heart failure patients.	√	√	√	√

*Note*: ED, emergency department; HF, heart failure; CNS, clinical nurse specialist; ANP, advanced nurse practitioner; PA, physician.

### 3.2 Advanced Clinical Practice

In recent years, several studies have highlighted the crucial role of advanced clinical practice (ACP), especially in the fields of cardiology and heart failure. ACP is emerging as a key element in the management of heart failure, improving the effectiveness of care and providing more coordinated and patient‐centered assistance. In this context, ACP refers specifically to the advanced nursing role, which may differ from the broader, multiprofessional meaning used in countries like the UK. Practical and clinical nurse specialists demonstrate the necessary skills to address the complexity of patients and ensure optimal clinical outcomes. An early study (Boulton et al. 1997) showed that clinical nurse specialists are capable of performing diagnostic cardiac catheterizations safely and effectively, producing high‐quality images without fatalities and with shorter procedure times. This not only highlights their competence but also their potential to improve efficiency in clinical practices. Meanwhile, another study (Albert et al. 2008) analyzed the impact of APNs and PAs in the administration of therapies for patients with heart failure. These results showed that having more than two APNs or PAs in a practice is associated with greater adherence to guidelines, suggesting that adequate professional support can significantly enhance clinical outcomes. Care coordination was further emphasized by Daniels (2014), who documented how a CNS can facilitate communication among various specialists, ensuring positive outcomes for mothers and infants. This multidisciplinary approach has proven effective in reducing confusion and improving the overall patient experience. Recent studies (King‐Dailey et al. 2022; O'Toole et al. 2019) have confirmed high patient satisfaction in services led by APNs, with timely diagnoses and a decrease in unnecessary admissions. This underscores how patient‐centered care and active support from nurses can significantly improve outcomes. Finally, Connolly et al. (2021) explored the innovative role of CNSs in interventional cardiology, highlighting how their presence as second operators in complex procedures contributes to ensuring continuity of care, improving process efficiency and reducing patient wait times.

### 3.3 Leadership

The results of several studies clearly highlighted the importance of leadership in ACP, especially in contexts related to cardiology and heart failure. Leadership not only influences the quality of care provided but also shapes team dynamics and enhances the overall patient experience. Boulton et al. (1997) indicated how CNSs, with appropriate training, are capable of performing complex procedures such as diagnostic cardiac catheterizations. This demonstrates that strong and well‐trained leadership is essential to ensure the safety and effectiveness of care. Kilpatrick et al. (2012) emphasized that leadership and the delegation of authority have a significant impact on the work of practical nurses. A work environment where leadership is clear enables nurses to better adapt to patient needs, effectively managing their workload and thereby contributing to the overall effectiveness of the team. In another study, Daniels (2014) underscored the crucial role of the clinical nurse specialist in coordinating care for complex patients, such as those with cardiac arrhythmias. This professional facilitates communication among different specialists, creating a collaborative context that not only improves the patient experience but also clinical outcomes. Similarly, Stuart (2017) highlighted how advanced practice nurses contribute to stabilizing work teams, particularly in environments where medical staff frequently changes. However, there is also concern that this role may lead to a loss of competencies among physicians, emphasizing the importance of clarifying roles and supporting teamwork. Finally, Connolly et al. (2021) demonstrated that, in the context of interventional procedures, clinical nurse specialists can improve process efficiency and reduce wait times. This underscores how active leadership from nurses not only fosters continuity of care but also promotes a more harmonious workflow within the team.

### 3.4 Facilitation of Education and Learning

The education and continuous learning of ANPs and CNSs are essential to ensure high‐quality care, particularly in the context of cardiology. Various studies highlight how interdisciplinary collaboration and specialized education can significantly enhance clinical practice. For instance, two studies (Daniels 2014; Griffiths 2006) emphasized how CNSs play a crucial role in coordinating multidisciplinary care, improving communication and patient outcomes, as well as leading interdisciplinary teams in managing clinical practices through the implementation of protocols and new therapies, thereby facilitating learning not only for nursing staff but also for other healthcare team members. Additionally, the adequate trained CNSs are able to perform complex interventions safely and effectively, achieving optimal diagnostic results and more efficient procedure times(Boulton et al. 1997). The complications encountered were minimal and manageable, confirming the feasibility of this approach in clinical practice (*p* < 0.05). This study underscores how specific training enhances practical skills. Two studies (Connolly et al. 2021; Johnson 2011) examined the role of ANPs in interventional cardiology, showing how good training improves continuity of care and efficiency, highlighting the value of interdisciplinary collaboration and offering high‐quality care, ensuring greater professional satisfaction and demonstrating how education continues to positively influence outcomes for these patients. Meanwhile, the study by O'Toole et al. (2019) recorded high levels of patient satisfaction in clinics led by ANPs, confirming that adequate training leads to faster diagnoses and reductions in admissions. Similarly, a study (Albert et al. 2008) found that practices with more than two APNs and PAs showed greater compliance with cardioverter‐defibrillator (ICD) implantation or for education on heart failure individuals (*P* < 0.01). On the other hand, the study by Kilpatrick et al. (2012) highlights how a heavy workload for ANPs affects their capacity for development and skill enhancement, emphasizing the importance of the work environment. Finally, a study (Houle et al. 2011) demonstrated that educational programs combined with socio‐cognitive support improve physical activity and quality of life for patients, indicating that education can have direct effects on health. Similarly, Charteris and Pounds (2020) reported that a quality improvement project increased patient satisfaction and their ability to self‐manage, underscoring the importance of educational support. Both studies highlight how education and structured support can improve patient outcomes, emphasizing the crucial role of well‐designed programs in enhancing health management and well‐being.

### 3.5 Evidence, Research, and Development

Numerous studies have highlighted the critical role of APNs in conducting research and developing evidence‐based practices in cardiology. For instance, research by Connolly et al. (2021) demonstrated that the involvement of specialized nurses in clinical trials, such as those related to innovative devices like the MitraClip for the treatment of mitral valve disease, not only enhances clinical practice but also improves therapeutic efficacy and continuity of care. These nurses are not only participants in clinical studies but are also engaged in patient education and the management of data collected during trials, thereby optimizing the overall care process.

Advanced practice nurses go beyond participating in research; they often lead projects aimed at addressing care gaps. For example, Ingram et al. (2017) showed that the role of the APN in cardiology improves the identification of patients with stable coronary artery disease and accelerates diagnostic timelines through effective triage and timely management of requests. This approach has led to improvements in patient satisfaction and service efficiency.

Heart failure management is another area where nursing research has made significant strides. King‐Dailey et al. (2022) demonstrated that the use of telemedicine, particularly through the involvement of nurse practitioners (NPs) in heart failure clinics, has reduced hospitalization rates and improved access to care for patients with chronic conditions. Research in this area continues to expand, with particular focus on the use of remote monitoring devices and telemanagement, which have become essential tools in enhancing patients’ quality of life and reducing pressure on the healthcare system.

Evidence from large‐scale studies and clinical practices has shown that APNs are effective in reducing healthcare costs, improving treatment adherence, and, most importantly, optimizing patient flow. Studies such as that by O'Toole et al. (2019) confirmed that advanced nursing management in patients with acute coronary syndrome not only improves clinical outcomes but also enhances patient satisfaction, underscoring the importance of innovative, evidence‐based care models.

Research continues to demonstrate that the integration of APNs in cardiology care not only optimizes resources but also provides continuous care that is crucial for patients with complex cardiovascular conditions.

### 3.6 Postgraduate Degrees and Educational Programs in Cardiology

The figure of the APN, as can be seen from the literature review, is continuously expanding internationally. In Europe, the APN is present in many countries: over 57% of EFN members reported that APNs are already established in their respective countries (De Raeve et al. 2024). Several studies (*n* = 9) addressed the issue of education and training pathways. Educational and training pathways for APNs in cardiovascular care reveal varied approaches across countries, with some similarities noted by different authors (Table 2). Connolly et al. (2021) describe the United Kingdom's requirement for APNs to hold at least a bachelor of science, postgraduate diploma, or master's degree. In addition, specific training under a consultant's supervision is required to obtain independent consent privileges, following a hands‐on model where APNs participate in procedures directly alongside consultants. This collaborative approach is also reflected in Boulton et al. (1997), who highlight that UK APNs gain cardiac expertise through ward‐based training and work in cardiac catheterization labs. In Canada, two studies (Kilpatrick et al. 2012; Griffiths 2006) note that a Master of Science degree is a common requirement, with a focus on ward‐based cardiac experience. Ingram (2014) specifies that in Ireland, APNs receive a master's degree with additional training in procedural site preparation and role‐specific job descriptions. In the United States, Johnson (2011) outlines the need for postgraduate medical education, while Albert et al. (2008) report specialized training in heart failure (HF) care, and Daniels (2014) mentions advanced training in high‐risk obstetric care.

**TABLE 2:** Training and educational pathway.

Author, year	Country	Education	Training
Boulton et al., 1997	UK		Ward‐based cardiac nursing Experience working in cardiac catheterization laboratories
Griffiths, 2006	Canada	Master of Science degree	NR
Nancy et al., 2010	USA		HF‐specialized training
Johnson, 2011	USA	Postgraduate medical education	NR
Kilpatrick et al., 2012	Canada	Master of Science degree.	NR
Daniels, 2014	USA		Advanced training in high‐risk obstetric care
Ingram, 2014	Ireland	Master of Science degree	Specific training in site preparation and job description creation
Connolly L et al., 2021	UK	Bachelor of Science Postgraduate Diploma Master of Science degree.	Specific training and observation by a consultant before gaining the ability to independently obtain informed consent Hands‐on training approach was adopted, where the CNS participated directly in all procedures, working closely with the consultant.

*Note*: NR = Not reported.

The diverse requirements across these countries, documented by multiple authors, underline a shared goal of fostering high procedural competence among APNs, albeit through varied national pathways and training methods.

In addition, research was conducted regarding the recognition and the role of the APN in cardiology, the certifications currently available and the associations related to them, recognized at the international level.

The search for evidence on cardiology specialization pathways, certification pathways, and professional associations identified nine countries (the USA, England, Canada, Australia, Ireland, Spain, the Netherlands, Finland, and Norway) where the APN in cardiology is a certified professional and regulated by nursing associations. The results obtained describe a large variability in the specialization title: a total of 8–10 post‐basic qualifications or courses were identified in Table 3. In the United States, nurses specializing in cardiology can obtain certification through the American Association of Critical‐Care Nurses (AACN) or the American Academy of Nursing Certification (AANC), both of which are permanently valid. In England, the “Cardiac Care Specialist” qualification is obtained through a one‐year postgraduate professional diploma course, which requires additional updates to remain active; it is supported by associations such as ESC, ACNAP, and BANCC. In Canada, the title “Cardiovascular Nurse Specialist” is certified by the Canadian Cardiovascular Nurses Association (CCCN), with permanent validity. In Australia, nurses can become “Cardiac Care Nurses” by completing a one‐year postgraduate certification course recognized by the ACNC. In Ireland, the “Coronary Care Nurse” title is granted through the PGDip postgraduate diploma from the Royal College of Surgeons Ireland (RCSI), supported by the Irish Nurses Cardiovascular Association (INCA). In Spain and the Netherlands, the titles “Cardiology Clinical Nurse Specialist” and “Nurse Practitioner in somatic health care,” respectively, require completion of Master's programs, supported by the European Society of Cardiology, the Association of Cardiovascular Nursing and Allied Professions, and the respective national associations. In Finland, CNS complete a Master's degree of 1.5 to 3 years, supported by the Finnish Nurses Association (FNA). In Norway, specialization in Advanced Clinical Nursing is achieved through a Master's degree but lacks formal continuous certification, with support from the Norwegian Nurses Organization (NNO).

**TABLE 3:** CNS in cardiology: certifications and nursing associations.

Country	Specialization title	Certification/Training	Title validity (y/n)	Nursing association
United States	Cardiovascular CNS	(1) AACN certification (2) AANC certification	Y	AACN; AANC
England	Cardiac care specialist	Professional course Postgraduate diploma (1 year)	N Y	ESC; ACNAP; BANCC
Canada	Cardiovascular nurse specialist	CNA Certification in Cardiovascular Nursing	Y	CCCN
Australia	Cardiac care nurse	Postgraduate course (1 year) certification	Y	ACNC
Ireland	Coronary care nurse	Postgraduate Diploma PGDip (RCSI) Professional Certificate	Y Y	INCA
Spain	Cardiology clinical nurse specialist	Master Degree (1 year)	Y	ESC; ACNAP; SANC; AEEC
Netherlands	Nurse practitioner somatic health care	2‐Year dual Master of Advanced Nursing Practice (MANP) degree	Y	ESC; ACNAP; DNSR
Finland	Clinical nurse specialist	Master's degree (1 ½ to 3 years)	Y	FNA
Norway	Advanced clinical nursing	Master's degree	N	NNO

*Note*: ANCC, American Nurses Credentialing Center; AACN, American Association of Critical‐Care Nurses; BANCC, British Association for Nursing in Cardiovascular Care; RCSI, Royal College of Surgeons in Ireland; INCA, Irish Nurses Cardiovascular Association; SANC, Spanish Association of Nurses In Cardiology; CCCN, Canadian Council of Cardiovascular Nurses; ESC, European Society of Cardiology; ACNAP, Association of Cardiovascular Nursing and Allied Professions; ACNC, Australasian Cardiovascular Nursing College; DNSR, Dutch Nursing Specialist Register; FNA, Finnish Nurses Association; NNO, Norwegian Nurses Organisation.

These differences highlight how professional certification and recognition are shaped by national regulations and supporting associations: specifically, a lower level of education (e.g., a vocational course) corresponds to a negative APN validation. This aspect underscores the importance of international standards to facilitate equivalency of competencies in the cardiovascular field.

## 4 Discussion

The findings of this scoping review highlighted the crucial contribution of APNs in enhancing patient management through continuity of care, process efficiency, and increased patient satisfaction, an outcome consistent with those observed in other high‐complexity clinical areas. Our results align with previous evidence underscoring the importance of APNs across various clinical care settings, emphasizing the significance of their role in diverse healthcare contexts (Franziska et al. 2024). For instance, in oncology, the role of APNs is recognized as essential in chemotherapy management and nursing care (Blakely and Cope 2015).

Studies have shown that APNs, equipped with advanced skills and specialized training, can ensure better adherence to treatment protocols and foster more effective communication between patients and multidisciplinary teams (Glarcher et al. 2024; McCorkle et al. 2015). This ability to improve complex treatment management also reflects in the psychological support provided to oncology patients and their families, reducing stress associated with long‐term care and promoting a patient‐centered approach to treatment (Stein et al. 2022). In surgical contexts, APN involvement in preoperative and postoperative management has shown a positive impact on patient recovery times and a reduction in postoperative complications. Several studies have documented how APNs in general and orthopedic surgery improve wound management, reduce infection rates, and promote healing, often working closely with surgeons and other specialists to optimize care pathways (Assolari et al. 2024). APNs not only manage educational interventions and follow‐up, but they also play a proactive role in the early identification of complications, demonstrating a high degree of autonomy and decision‐making capability (Hollenbeck et al. 2023).

Another key finding concerns the contribution of APNs to care coordination and teamwork in multidisciplinary settings. Numerous studies have indicated that the presence of an APN facilitates communication and collaboration among specialists, reducing misunderstandings and enhancing the overall patient experience (Elizabeth 2023; Gleeson et al. 1990). For example, a recent study highlighted the value of the CNS's role in managing care for nutritional assessment, demonstrating the effectiveness of advanced nursing coordination in multidisciplinary patient management (Mancin et al. 2024; Starace et al. 2023). Additionally, Poghosyan et al. (2022) found that APNs play an important role in ensuring team stability, especially in high‐turnover environments, thereby stabilizing the workplace and improving continuity of care.

Regarding leadership, literature supports the idea that APNs in cardiology not only positively influence the quality of care but also play a vital role in shaping team dynamics and enhancing the patient experience. Boulton et al. (1997) showed that specialized nurses with appropriate training can take on leadership responsibilities in complex procedures, highlighting the importance of well‐trained leadership in ensuring the safety and effectiveness of care. Kilpatrick et al. (2012) emphasized how leadership and delegation of authority significantly impact practical nursing work, confirming that a work environment that values leadership enables nurses to better meet patient needs and effectively manage their workload.

Finally, continuous education has been identified as a critical factor for the success of APNs in cardiology. A previous study has shown how interdisciplinary collaboration and specialized training significantly enhance clinical practice (Kleinpell 1999). Griffiths (2006) and Daniels (2014) argue that the APN role, which coordinates multidisciplinary care and implements innovative protocols, is key not only in patient care but also in educating and guiding the healthcare team. Recent studies have also shown that specific training for APNs in cardiology leads to improved patient satisfaction, with more timely diagnoses and a reduction in unnecessary hospitalizations (Donald et al. 2014; Ordóñez‐Piedra et al. 2021). Although this scoping review explored all types of APNs, including Nurse NPs and CNSs, it should be noted that APNs working in cardiology are more likely to align with the CNS profile (Hamric et al. 2014). The findings from this review, demonstrating APNs’ involvement in clinical care, education, and research, are consistent with the International Council of Nurses’ (ICN) definition of an APN (ICN 2020). This further reinforces the central role of APNs in delivering high‐quality care and supporting complex care pathways in cardiology.

The findings of this review contribute to the growing body of evidence on the essential role of APNs in cardiology, suggesting that their presence and advanced competencies can significantly improve clinical outcomes, continuity of care, and process efficiency. These findings are consistent with other international research that confirms the importance of formal recognition and standardized training pathways to maximize the impact of APNs in nursing care (Htay and Whitehead 2021). However, variations in training requirements and certifications internationally indicate the need for standardization to facilitate competency equivalency and ensure uniform care levels on a global scale (Assolari et al. 2024).

### 4.1 Implications for Nursing and Health Policy

This scoping review highlights the importance of an expanded implementation of the APN role across various clinical contexts, extending beyond cardiology management. With advanced competencies and decision‐making capabilities, APNs are established as essential resources for enhancing the quality and continuity of care, yielding documented benefits for patients with complex and chronic conditions. Adopting advanced practice models could represent an effective solution to meet the increasing demand for integrated, personalized care across diverse clinical settings. Additionally, promoting specific training pathways and ongoing professional development for APNs would facilitate skill standardization, fostering a more efficient healthcare system oriented toward improved patient outcomes. From a health policy standpoint, integrating APNs into multidisciplinary teams would not only support treatment effectiveness but could also help alleviate the workload of other healthcare professionals, promoting resource optimization and cost reduction. A stronger focus on these aspects would additionally help ensure standardized competencies and equitable access to advanced care models.

### 4.2 Study Limitations

This study has certain limitations that should be considered. First, the review was conducted on a limited selection of studies, which may affect the representativeness and generalizability of the results. Moreover, including articles from specific healthcare settings or geographic regions may limit the applicability of the findings on an international scale, as advanced practice models can vary significantly between countries. Another limitation concerns the lack of longitudinal studies included in this review, which might have provided a more comprehensive understanding of the long‐term effects of APN interventions on patient outcomes. Additionally, the inclusion of all types of APNs, such as NPs and CNSs, while offering a comprehensive overview, may also introduce variability in role definitions and responsibilities that could affect the interpretability and generalizability of the results. This heterogeneity underscores the complexity of standardizing the APN role in cardiology and the need for greater clarity in professional definitions. Finally, the heterogeneity of APN competencies and responsibilities across clinical contexts makes it challenging to establish uniform standards, underscoring the need for further research to identify optimal strategies for APN integration within care teams.

## 5 Conclusions

This scoping review has highlighted the critical role of APNs in cardiology, confirming that integrating this professional figure is essential for enhancing the effectiveness, continuity, and quality of care for patients with cardiac conditions. APNs in cardiology play a critical role in facilitating timely interventions, particularly in time‐sensitive situations such as stroke thrombolysis, where rapid decision‐making and prompt action are essential for optimizing patient outcomes. APNs have demonstrated a significant impact in managing chronic and acute conditions, reducing exacerbations, and improving outcomes for patients with heart failure, arrhythmias, and other complex CVDs. Additionally, the high level of clinical expertise and decision‐making autonomy of APNs allows for more effective implementation of care protocols, optimizing intervention times and fostering integrated patient management.

The findings of this review suggest that strengthening the APN role in cardiology, supported by advanced, specialized training programs, could serve as a key strategy to address the complexities of cardiovascular care. In particular, standardizing competencies and educational pathways could ensure greater uniformity in practices and enhance outcomes globally, making APNs an even more effective resource within multidisciplinary cardiology teams. Finally, further studies should evaluate the long‐term impact of APNs on patient outcomes in cardiology to solidify the evidence base and inform healthcare policies that support the integration of this role.

## Author Contributions

MS: Conceptualization, methodology, writing original draft, review and editing, investigation, visualization; SM: Conceptualization, methodology, writing original draft, review and editing, investigation, project administration; VC: Writing original; NS: Review and editing; NS: Review and editing; GC: Review and editing; SMP: Review and editing, supervision; GF: Writing original draft, review and editing; supervision; ALC: Writing original draft, conceptualization, methodology, supervision. MS and SM provided an equal contribution as the first author in drafting the manuscript; GF and ALC provided an equal contribution as the last author. All authors read and approved the final manuscript.

## Conflicts of Interest

The authors declare no conflict of interest relevant to this article.

## Use of Artificial Intelligence

This manuscript is an original contribution, and the use of artificial intelligence was limited solely to the final language editing and grammar correction of the manuscript.

## Supporting information




**Supporting Table 1**: (Search strategy).
**Supporting Table 2**: (Characteristic of Included Studies).
