# Supplementary material for: Exploring the Role of Advanced Practice Nurses in Cardiology: A Scoping Review
Source: Int Nurs Rev. 2025 Jun 29;72(3):e70054. doi: 10.1111/inr.70054 (PMC12206687; doi:10.1111/inr.70054)
Supplement: Supplementary file 1 — Supporting Table 1: (Search strategy). Supporting Table 2: (Characteristic of Included Studies). [file INR-72-0-s001.docx]

**SUPPLEMENTARY FILES**

**TABLE S1.** Search strategy

| **ID** | **Cochrane Library** | **Number of articles found** |
| --- | --- | --- |
| #1 | “Clinical Nurse Specialist” OR “Advanced Practice Nurse” OR “Clinical Nurse Consultant” OR “Nurse Practitioner” OR “Advanced Practice Registered Nurse” OR “Specialist Nurse Practitioner” OR “Clinical Nursing Specialist” OR “Advanced Clinical Nurse” OR “Clinical Nurse Leader” OR “Clinical Nurse Educator” OR “Clinical Advanced Practice Nurse” OR “Clinical Nursing Consultant” | **Results** |
| #2 | “Cardiology” OR “Cardiac Procedures” OR “Cardiac Interventions” OR “Cardiac Procedure” OR “Cardiac Operations” OR “Cardiac Treatments”) |  |
| **#3** | **#1 AND #2** | **15** |
| **ID** | **PubMed** | **Results** |
| #1 | "Nurse Specialists"[Mesh] OR “Clinical Nurse Specialist” OR “Advanced Practice Nurse” OR “Clinical Nurse Consultant” OR “Nurse Practitioner” OR “Advanced Practice Registered Nurse” OR “Specialist Nurse Practitioner” OR “Clinical Nursing Specialist” OR “Advanced Clinical Nurse” OR “Clinical Nurse Leader” OR “Clinical Nurse Educator” OR “Clinical Advanced Practice Nurse” OR “Clinical Nursing Consultant” |  |
| #2 | (Cardiology [MeSH Terms]) OR (“Cardiology” OR “Cardiac Procedures” OR “Cardiac Interventions” OR “Cardiac Procedure” OR “Cardiac Operations” OR “Cardiac Treatments”) |  |
| **#3** | **#1 AND #2** | **288** |
| **ID** | **Cumulative Index of Nursing and Allied Health Literature (CINAHL)** | **Results** |
| #1 | “Clinical Nurse Specialist” OR “Advanced Practice Nurse” OR “Clinical Nurse Consultant” OR “Nurse Practitioner” OR “Advanced Practice Registered Nurse” OR “Specialist Nurse Practitioner” OR “Clinical Nursing Specialist” OR “Advanced Clinical Nurse” OR “Clinical Nurse Leader” OR “Clinical Nurse Educator” OR “Clinical Advanced Practice Nurse” OR “Clinical Nursing Consultant” |  |
| #2 | “Cardiology” OR “Cardiac Procedures” OR “Cardiac Interventions” OR “Cardiac Procedure” OR “Cardiac Operations” OR “Cardiac Treatments” |  |
| **#3** | **#1 AND #2** | **121** |
| **ID** | **EMBASE** | **Results** |
| #1 | 'clinical nurse specialist':ab,ti OR 'advanced practice nurse':ab,ti OR 'clinical nurse consultant':ab,ti OR 'nurse practitioner':ab,ti OR 'advanced practice registered nurse':ab,ti OR 'specialist nurse practitioner':ab,ti OR 'clinical nursing specialist':ab,ti OR 'advanced clinical nurse':ab,ti OR 'clinical nurse leader':ab,ti OR 'clinical nurse educator':ab,ti OR 'clinical advanced practice nurse':ab,ti OR 'clinical nursing consultant':ab,ti |  |
| #2 | 'cardiology':ab,ti OR 'cardiac procedures':ab,ti OR 'cardiac interventions':ab,ti OR 'cardiac practices':ab,ti OR 'cardiac operations':ab,ti OR 'cardiac treatments':ab,ti |  |
| **#3** | **#1 AND #2** | **223** |
| **TOTAL ARTICLES FOUND** | | **647** |
| **DUPLICATES** | | **93** |
| **AFTER TITLE AND ABSTRACT READ** | |  |
| **AFTER FULL TEXT READ** | |  |

**TABLE S2.** Charatcteristic of Included Studies

| Characteristic | Frequency (n=15) | Percentage |  |
| --- | --- | --- | --- |
| Publication year  1997  2006  2010  2011  2012  2014  2017  2019  2020  2021  2022 | 1  1  1  1  2  3  2  1  1  1  1 | 6.7%  6.7%  6.7%  6.7%  13.4%  20.0%  13.4%  6.7%  6.7%  6.7%  6.7% |  |
| Geographical distribution | | | |
| Western Countries  USA  Canada  United Kingdom  Ireland | 15  5  3  3  4 | 100%  33.3%  20.0%  20.0%  26.7% |  |
| Type of studies | | | |
| Primary  Observational/descriptive study  Qualitative study  Cross-sectional study  Randomized Control Study  Case study  Cohort study  Secondary (literature review)  Narrative Review | 13  6  1  2  1  2  1  2  2 | 86.7%  46.1%  7.7%  15.4%  7.7%  15.4%  7.7%  13.3%  100% |  |
| Topic | | | |
| CNS support in therapy  CNS role in management of heart failure  Multidisciplinary nursing collaboration  CNS/APN role  CNS role in Discharge protocols | 2  4  3  5  1 | 13.3%  26.7%  20.0%  33.3%  6.7% |  |

Note: CNS= Clinical Nurse Specialist; APN=Advanced Practice Nurse
